# Supplementary material for: A high-resolution real-time quantification of astrocyte cytokine secretion under shear stress for investigating hydrocephalus shunt failure
Source: Commun Biol. 2021 Mar 23;4:387. doi: 10.1038/s42003-021-01888-7 (PMC7988003; doi:10.1038/s42003-021-01888-7)
Supplement: Supplementary file 2 — Description of Additional Supplementary Files [file 42003_2021_1888_MOESM2_ESM.pdf]

Description of Additional Supplementary Files

File Name: Supplementary Data 1

Description: Source data for TIR-FM and ELISpot assays
